# Hexamodal awake brain mapping (language, sensorimotor, ictal, visual, auditory) for multilobar resection in a dominant hemisphere parieto-fronto-temporo-occipital cortical malformation with drug-resistant epilepsy

**DOI:** 10.3171/2024.10.FOCVID24111

**Published:** 2025-01-01

**Authors:** Lokesh V. Dasarathan, George C. Vilanilam, Ankush R. Parate, Revikrishnan Sreekumar, Smita Vimala, Manikandan Sethuraman, Ramshekhar N. Menon, Ashalatha Radhakrishnan, Karamala Y. Manisha, Nandana Jayakumari, Rajalakshmi Poyuran, Nandini S. Nair, Anees Chembakodan, Manju P. Mohan, Vipina V. Padmakumari, Nayana L. Nair, Rakesh Vashishta, Chandrasekharan Kesavadas, Bejoy Thomas, Krishnakumar Kesavapisharady

**Affiliations:** 1 Department of Neurosurgery,; 2 Department of Neuroanaesthesia, and; 3 R. Madhavan Nayar Center for Comprehensive Epilepsy Care, Sree Chitra Tirunal Institute of Medical Sciences and Technology, Thiruvananthapuram, Kerala, India

**Keywords:** brain mapping, cortical mapping, hexamodal, eloquent cortex epilepsy

## Abstract

Surgically remediable epilepsy of the eloquent brain poses the added challenge of preserving function while curing disease. Long-standing epileptogenic lesions have tenacious seizure networks and significant functional reorganizations. Large multilobar lesions may involve multiple functional areas, thereby challenging the limits of functional brain mapping. In this video, the authors describe a dominant hemisphere fronto-parieto-temporo-occipital malformation of cortical development, involving the language, sensorimotor, and visual cortices, placing multiple eloquent cortical and subcortical regions at risk. They illustrate the technique of hexamodal mapping/monitoring involving the language sensorimotor areas, optic radiation, auditory pathway, and ictal irritative zone for a multilobar resection, with good seizure outcome and function preservation.

The video can be found here: https://stream.cadmore.media/r10.3171/2024.10.FOCVID24111

## Transcript

Hexamodal awake brain mapping for multilobar resection in a dominant hemisphere cortical malformation with drug-resistant epilepsy.

### 0:28 Clinical History.

A 21-year-old male presented with seizures of 15 years’ duration with two semiologies and three to four episodes per month not controlled on five anticonvulsants. He was right-handed with no focal neurological deficits, and neuropsychology assessment was normal.

### 0:45 Preoperative Evaluation.

Video EEG showed left temporocentric spikes. The MRI showed a large left temporo-parieto-occipital cortical malformation, with fMRI showing language and faciobrachial function localized within the lesion. DTI and synthetic MRI helped to delineate the gray–white matter better. The abnormal cortical architecture was delineated better by a synthetic MRI. This video clip shows that the patient had no focal neurological deficits. Preoperative visual assessment was normal.

### 1:23 Surgical Plan and Theater Setup.

We planned for an awake multilobar resection with hexamodal mapping considering the at-risk language, motor, sensory, visual, and auditory functional areas and subcortical tracts. We also mapped the ictal irritative zone using intraoperative electrocorticography.^1^ Here are the language assessment charts and the schematic operation theater layout. A scalp block was given and the pin site infiltrated. The patient was positioned supine, neutral, with 30° rotation. The scalp EEG electrodes and peripheral neuromonitoring electrodes were placed. Monopolar, bipolar, and suction stimulators were used. The hexamodal monitoring parameters are enumerated in this table.^2,3^ The monitored anesthesia care included moderate sedation initially, awake monitoring stage, and finally moderate sedation again. Bispectral index (BIS) for the depth of sedation. Ear plug stimulator used for AEP.

### 2:20 Craniotomy and Hexamodal Mapping.

The incision was planned using neuronavigation guidance. Baseline VEP obtained. A left fronto-temporo-parietal craniotomy was done, and the dura was opened based on the superior sagittal sinus. Electrocorticography recordings were obtained from the surface using a 20-contact grid, and the spiking zones were mapped. SSEP phase reversal technique was done to identify the central sulcus. No afterdischarges were noted after cortical stimulation in the 20-contact grid. Motor and sensory mapping was then done using bipolar stimulation starting with 1 mA and gradually increased up to 8 mA. Mapping of the hand, face, and lip area was done with serial increase in current intensity. ECoG was also simultaneously looked for any afterdischarges. A positive mapping was obtained in the hand area at 8 mA and noticed in the form of a clinical twitch and EMG-positive responses. Next, we attempted to map the face area, and we got a negative mapping as per the protocols. The leg area mapping was also negative despite increasing the current as per protocol. After negative mapping of the face and the lip, language mapping was then done. This was done in the patient’s native Indian language, Malayalam. This is the schema of the hexamodal mapping.

### 4:07 Speech Assessment (assessment in native Indian language, Malayalam).

Where are you from?

Who stays with you in your home?

What is your father’s name?

Address of your home?

Identify this number.

### 4:25 Resection Commenced.

The pia arachnoid was dissected open in the superior parietal lobule to begin the resection. The resection was begun in the least functional areas that were negatively mapped in the superior parietal lobule. Piecemeal resection using suction and CUSA was done. Intervascular subpial resection protecting the prominent vein of Trolard was the mainstay of the piecemeal resection. Language assessment included naming, oral comprehension, repetition, semantic and phonemic fluency, and reading words, phrases, and numbers.

### 5:01 Speech Assessment (assessment in native Indian language, Malayalam).

Repetition.

Repeat: flower, picture, 45, Thiruvananthapuram Medical College, [language twisters in Malayalam: "All that glitters is not gold"].

Count from 1 to 30.

### 5:44 Subcortical Mapping.

Monopolar suction stimulator was used for subcortical mapping of the arcuate fibers. Bipolar probe was then next used for stimulation and mapping of the postcentral gyrus. The negatively mapped areas were subpially resected in a piecemeal manner. Subcortical motor mapping was done using a suction stimulator, and subpial resection was gently done. Underneath the vein of Trolard, a rim of parenchyma was left for support. Subcortical mapping was negative in the deep subcortical white matter. The negative mapping helped us to do a more aggressive resection in the anatomical postcentral gyrus. As the resection extended into the supramarginal, angular gyrus, we restarted the language mapping.

### 6:40 Speech Assessment (assessment in native Indian language, Malayalam).

Object identification, picture naming, color naming.

What is this called?

What is this color?

### 7:03 Subpial Intervascular Resection.

Subcortical mapping was further continued using the suction stimulator, and gentle subpial intervascular resection was done preserving the vein of Trolard.^4^ The resection of the large cortical malformation was extended further gyrus by gyrus after negative cortical mapping and subcortical negative mapping. Further piecemeal resection of the postcentral gyrus was done, preserving a bridge of tissue under the vein of Trolard. As the resection proceeded into the supramarginal and angular gyrus, the language mapping was restarted.^5^

### 7:48 Speech Assessment (assessment in native Indian language, Malayalam).

Object identification, fluency, identify alphabets, complex sentence framing.

Where is the matchbox in the picture?

Where is the pencil?

Where is the cup?

Identify the letter B.

Identify the letter J.

Identify the letter M.

### 8:30 Postresection ECoG.

Postresection ECoG showed persistent spikes only in the posterior temporal cortex, and further tailored resection was done. However, it was limited due to speech repetition being affected during language mapping. Mild hand weakness during motor mapping resection also precluded further additional resection in the motor cortex.

[Resection attempted with language mapping.]

### 8:53 Speech Assessment (in native Indian language, Malayalam).

Complex repetition.

Repeat: flower, 45.

[Repetition impaired.]

### 9:16 Postresection Status.

After the additional tailored resection, the ECoG showed reduction of spikes and hemostasis was achieved. The AEP and VEP also showed no postoperative changes. Bone flap was replaced, and the wound was closed in layers. The patient recovered well with an Engel class I outcome and mild right hemiparesis. The postoperative MRI showed near-total excision of the lesion with a good visual profile. Histology showed a type IIB dysplasia, and this is a schematic view of the resection cavity with reference to the white matter tracts.^6^

### 9:50 Discussion and Conclusions.

Multimodality brain mapping helps to accurately delineate the functional shifts^1–4^ and offer extended safe resections, thus balancing functional preservation versus adequacy of resection.^6–8^ To conclude, multimodality mapping of functional areas guides resection of multilobar eloquent epileptogenic lesions while preserving optimal function and extirpating epileptogenic zones and networks.

## Disclosures

Dr. Thomas reported grants from DST, Government of India; and ICMR, Government of India, outside the submitted work.

## Author Contributions

Primary surgeon: Vilanilam. Assistant surgeon: Dasarathan, Parate. Editing and drafting the video and abstract: Vilanilam, Dasarathan, Parate, Sreekumar, Sethuraman, Menon, Radhakrishnan, Manisha, Jayakumari, Poyuran, Kesavapisharady. Critically revising the work: Vilanilam, Dasarathan, Parate, Vimala, Sethuraman, Menon, Radhakrishnan, Kesavadas, Kesavapisharady. Reviewed submitted version of the work: Vilanilam, Menon, Kesavadas, Thomas, Kesavapisharady. Approved the final version of the work on behalf of all authors: Vilanilam. Supervision: Vilanilam, Menon, Kesavadas. Intraoperative neurophysiological monitoring: Sreekumar, Nair, Chembakodan. Intraoperative speech assessment: Mohan, Padmakumari, Nair. Intraoperative neuronavigation: Vashishta. Pathology: Poyuran. Video recording: Nair, Chembakodan, Mohan, Vashishta.

## Supplemental Information

### Patient Informed Consent

The necessary patient informed consent was obtained in this study.
